# 

**Published:** 1863-01

**Authors:** 


### CONTENTS.

| ART. | | PAGE |
|---|---|---|
| I. | On the Nature of Volition. By J. Lockhart Clarke, F.R.S. | 1 |
| II. | On Cooling of the Body after Death. By Benjamin W. Richardson, M.A., M.D. | 24 |
| III. | Free Trade in Medicine | 36 |
| IV. | Mania Ephemera. By J. Crichton Browne, M.D. | 45 |
| V. | Morell's Inductive Mental Philosophy | 59 |
| VI. | Gratuitous Medical Services | 82 |
| VII. | On a New Theory of Vision. By J. Alexander Davies | 92 |
| VIII. | On Morbid Impulse. By W. Carmichael McIntosh, M.D. | 101 |
| IX. | The Sickness and Mortality of the Federal Volunteers | 123 |
| X. | On the Unconscious Life of the Soul | 132 |
| XI. | Dangerous Classes | 136 |
| XII. | Medical Certificates in Lunacy | 154 |
| XIII. | Gossip | 161 |
| | Literary Retrospect | 172 |
| | Foreign Medico-Psychological Literature | 183 |
| | Sir Benjamin Brodie, Bart. | 192 |

No. X. will be published on the 1st of April.

